# Comparative analysis of the lethality pattern of Covid-19 and the response of BRICS countries to the pandemic in the context of multilateralism: An ecological study

**DOI:** 10.1371/journal.pone.0332883

**Published:** 2025-09-30

**Authors:** Thalyta Cássia de Freitas Martins, Adelyne Maria Mendes Pereira, Cristiani Vieira Machado, Carlos Machado de Freitas, Raphael Mendonça Guimarães

**Affiliations:** 1 National School of Public Health, Oswaldo Cruz Foundation. Road Leopoldo Bulhões, Manguinhos, Rio de Janeiro, Brazil,; 2 Estácio de Sá University, Institute of Medical Education, Rio de Janeiro, Brazil; The University of Edinburgh, UNITED KINGDOM OF GREAT BRITAIN AND NORTHERN IRELAND

## Abstract

**Background:**

This article analyzes the pattern of lethality due to Covid-19 in the BRICS countries and its conditioning factors, analyzing its temporal evolution, as well as analyzing the response given by the grouping to the health crisis as a multilateral forum.

**Methods:**

This ecological time-series study assessed the lethality due to Covid-19 in the BRICS countries from March 2020 to May 2023. Using the Joinpoint method, and the temporal trend analysis of the lethality rates by segmented regression for each BRICS country.

**Results:**

In Brazil, the highest case fatality rate (8%) occurred in phase 1 (from April 27, 2020 to November 10, 2020), despite the peak in cases (1.2 million/week) in phase 3 (from December 31, 2021 to April 21, 2022). In Russia, phase 2 (from September 20, 2020 to March 7, 2021), which had the lowest incidence, had the second highest case fatality rate (4.5%). In India, the peak in cases in phase 2 (from March 13, 2021 to July 19, 2021) did not coincide with the peak in case fatality in the same phase. In China, the highest case fatality rate (12%) occurred in phase 1 (from March 10, 2022 to March 24, 2022), not reflecting the incidence. In South Africa, at the end of 2022, the fatality rate achieved was around 4.5%.

**Discussion:**

The lethality of Covid-19 among BRICS countries showed differences related to failures in testing capacity, death information systems and access to vaccines. The group did not achieve the expected performance at an extremely opportune moment to demonstrate to the world their differential as representatives of emerging powers.

## Introduction

The Covid-19 pandemic has affected all countries in the world, generating a major impact on health systems, economic, social and political systems, creating challenges for global health sovereignty and requiring a shift from an individual response to a multilateral approach based on health diplomacy [1]. The BRICS countries (Brazil, Russia, India, China and South Africa) especially India, Russia and Brazil were some of the countries with the highest number of Covid-19 cases in the world, in addition, most of the variants were initially registered in BRICS countries (B.1.351 – Beta in South Africa, P.1 – Gamma in Brazil, and B.1.617.2 Delta in India) [2]. In August 2023, the BRICS were responsible for more than a quarter of the global total of cases and deaths from Covid-19 (27.19% and 26.77% respectively) [3].

BRICS has stood out as an alternative for the reconstruction of multilateralism, promoting greater democratization in decision-making and the renewal of Global Governance [4]. In April 2020, the group declared that the fight against Covid-19 was on the Bloc’s agenda, which was reaffirmed during the Extraordinary Conference of Foreign Ministers on Covid-19 held in the same month. At the time, the bloc’s countries agreed to intensify information sharing and research collaboration, especially in the field of vaccine production, and committed to strengthening multilateralism [5]. However, the joint production of an immunizer by BRICS was not carried out due to the late inauguration of the BRICS Vaccine Production and Development Center, in March 2022, two years after the pandemic, online. In addition, there were internal bureaucratic obstacles to the group, such as the challenges faced by each country in managing the health crisis. In this regard, it is noteworthy that all BRICS countries, with the exception of China, adopted social distancing measures that were limited, flexible, and under decentralized coordination. Brazil, India, and South Africa demonstrated weak social assistance measures, which are essential to sustaining social distancing measures. Regarding vaccination, Brazil, India, and South Africa began their campaigns late; Russia, India, and South Africa had vaccination coverage (complete schedule) of less than 70% of the population in December 2022, and all five countries experienced vaccine hesitancy. [6–8].

It is important to highlight the urgent need to establish a new global governance architecture, supported, among other premises, by the restructuring of multilateral institutions as a safe means of facing common threats such as Covid-19 [9]. In this context, given the enormous potential of BRICS cooperation in the current scenario of multilateralism, the following research questions emerged: Was there convergence in the pattern of lethality due to Covid-19 among the BRICS countries and in the group’s response to the disease as a multilateral institution? In this sense, this article aims to describe the pattern of lethality due to Covid-19 in the BRICS countries and its conditioning factors, analyzing its temporal evolution, as well as analyzing the response given by the group to the health crisis as a multilateral forum.

## Materials and methods

### Study design and data source

This is an ecological time series study that assessed the lethality of Covid-19 in the BRICS countries (Brazil, Russia, India, China and South Africa), from January 2020 to May 2023. This interval corresponds to the entire period in which Covid-19 was considered a pandemic by the World Health Organization plus the beginning of fisrt year of pandemic, as we have some cases described as COVID-19 in some countries due to backward surveillance. Data on new cases, new deaths and lethality from Covid-19 were obtained from the Our World in Data platform (https://ourworldindata.org/), a digital publication specialized in exposing empirical research and analytical data on changes in quality of life conditions around the world. The dependent variables were the incidence, mortality and fatality rates for Covid-19 and the independent variable was time.

### Data analysis

Initially, we considered the absolute number of new daily cases of covid-19 in the five BRICS countries. Since the analysis considered a short period, we assumed that the population variation was not large and, therefore, it was possible to use absolute data to describe the historical series in each country [10]. We emphasize that this strategy is only possible because we do not compare the magnitude of incidence between countries, but rather for each country individually. By creating the historical series for each country, we were able to describe the phases of the pandemic in each country. We consider a phase to be any period in which there was a notable increase in cases, a peak and a subsequent decline, until there was a resumption of the increase in new cases again. Once we classify the phases in each country, we check the behavior of the fatality rate in each of them. It is noteworthy that the phases reflected specific contexts of each BRICS country, since mortality was used as an analysis criterion, whose data has a smaller time lag and quality in the death certificate registration, as well as a greater probability of testing, as these are usually symptomatic cases, being a criterion used by the Ministries of Health of the countries studied and by the World Health Organization for epidemiological analysis.

The lethality was calculated by dividing the number of confirmed deaths from Covid-19 in the last seven days in the population residing in the country (numerator) divided by the total number of Covid-19 cases reported in the last seven days, multiplied by 100. The first stage was a visual inspection of the historical lethality series, in order to verify discrepancies between countries, and to verify whether there was similarity in trends between the incidence and lethality series. Next, we try to identify the inflection points within each phase that created breaks in the temporal trend, thus starting a new time segment with a different trend from the previous one. This technique is known as segmented regression.

The temporal trend analysis of lethality by segmented regression for each BRICS country was carried out using the Joinpoint method, through which we sought to identify changes in level and trend in the historical series in the countries studied [11]. To this end, this model assumes a linear trend between the inflection points (*joinpoints*). Thus, whenever there is a significant change between a junction of points and the next point, over time, we assume that there is an inflection point there. From this point a new regression line is started. We define the junction point model for the observations, (x₁, y₁),…, (xₙ, yₙ), onde x₁ ≤ ⋯ ≤ xₙ without loss of generality, as:


$$\text{E}[\text{y}/\text{x}=\beta_{\text{0}}+\beta_{\text{1x}}+\delta_{\text{1}}(\text{x}-\tau_{1}{)}^{+}+\cdots +\delta_{k}(\text{x}-\tau_{k}{)}^{+}$$

where τ_k_’s are the joinpoints unknown and a^+=a for a > 0 e 0 otherwise [8]. To ensure the assumption of homoscedasticity, we use Poisson distribution parameters with robust variance. To estimate the *Annual Percentage Change* (APC), we use the following model:


$$\text{log}(\text{Yx})=\beta_{\text{0}}+\beta_{\text{1x}}$$

where log (Yx) is the natural logarithm of the rate in the year x.

Então, o APC de ano x para o ano x + 1 é:


$$\text{APC}=\frac{{\text{e}}^{\beta_{\text{0}}+\beta_{\text{1}}(\text{x}+\text{1})}-e^{\beta_{\text{0}}+\beta_{\text{1x}}}}{{\text{e}}^{\beta_{\text{0}}+\beta_{\text{1x}}}}\times \text{100}=({\text{e}}^{\beta_{\text{1}}-\text{1}})\times \text{100}$$

The 95% confidence interval for the APC is defined by: (APCL, APCU), where:


$$\text{APCL}=({\text{e}}^{\text{log}(\text{APC}+\text{1})-\text{1},\text{96}\sqrt{}\text{wx}\text{2}\sigma \text{x}\text{2})}-\text{1})\times \text{100};\text{APCU}=({\text{e}}^{\text{log}(\text{APC}+\text{1})+\text{1},\text{96}\sqrt{}\text{wx}\text{2}\sigma \text{x}\text{2})}-\text{1})\times \text{100}$$

being σ^2^ x the estimate of the variance of b_x_ obtained from the adjustment of the model of *joinpoint*.

Finally, we analyzed the correlations between the incidence and mortality rates of Covid-19 using Pearson’s correlation test [12]. The Kolmogorov-Smirnov and Shapiro-Wilk normality tests were performed to demonstrate the non-normal distribution of incidence and mortality with p-value < 0.05 in both tests. Statistically significant results (p-value < 0.05) of Pearson correlation were classified into five categories: very weak (0.00–0.19); weak (0.20–0.39); moderate (0.40–0.59); strong (0.60–0.79); very strong (0.80–1.00) [13].

We perform the selection of the number of inflection points using the *software Joinpoint Regression Program* (*Statistical Research and Applications Branch, National Cancer Institute*, Bethesda, Maryland, EUA), version 4.9.1.0., April of 2022 (https://surveillance.cancer.gov/joinpoint/), through Monte Carlo permutation tests [14]. We considered a significance level of 5%. Correlation analyses were performed using the program R version 4.2.1; and the graphs, built using the Excel program.

This study was conducted exclusively with publicly available secondary data, in accordance with the ethical conduct in research involving human beings, as set out in Resolution No. 466/2012 of the National Health Council [15]. Therefore, assessment by a Research Ethics Committee was not necessary.

## Results

**Fig 1:**
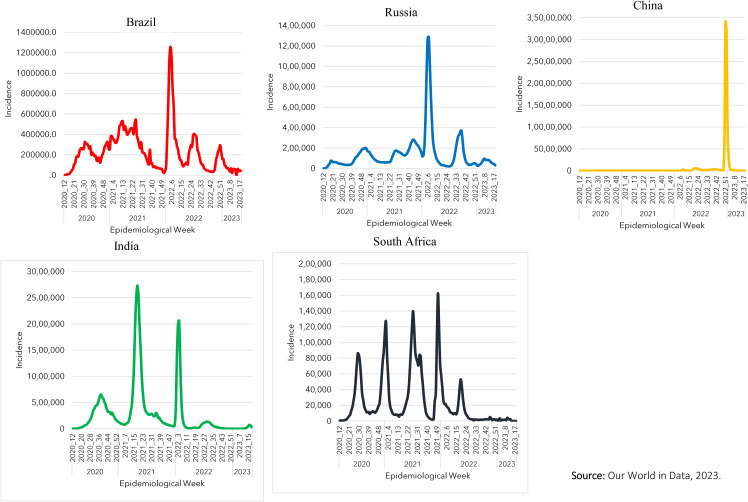
Time series of Covid-19 incidence in BRICS countries (2020-2023). **Source:** Our World in Data, 2023.

The countries presented very different historical series of Covid-19 incidence (Fig 1). In the case of Brazil, the second pandemic phase, which occurred between epidemiological weeks 47 of 2020 and 49 of 2021, was the longest, and the third, recorded between epidemiological weeks 2 and 17 of 2022, was the most severe, reaching a weekly average of over 1.2 million cases. In Russia, the most significant pandemic phase, which occurred between epidemiological weeks 02 and 17 of 2022, recorded more than 1.2 million new weekly cases. In India, the second and third pandemic phases, which occurred between epidemiological weeks 09–29 of 2021 and 02–12 of 2022 respectively, were the most severe, recording an average of more than 2.5 million cases. In China, the first two pandemic phases that occurred between 2020 and 2022 did not record a significant number of Covid-19 cases in the country. The third phase, however, which occurred between epidemiological weeks 47 of 2022 and 05 of 2023, recorded a weekly average of around 35 million new cases. In South Africa, the third wave recorded between epidemiological weeks 19 and 39 of 2021 is noteworthy, where there was a combination of two contiguous waves and immediately afterwards the fourth wave, which occurred between epidemiological weeks 44 of 2021–17 of 2022, which recorded the highest average of weekly cases in the country (160,000).

**Fig 2:**
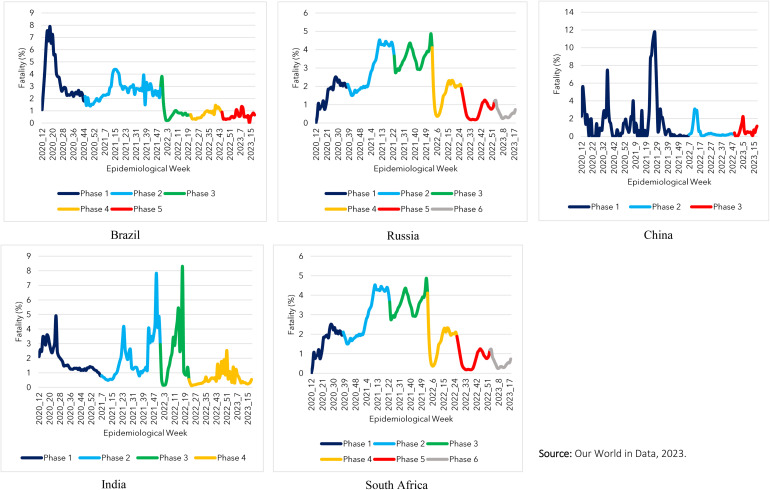
Fatality by phase of the pandemic in BRICS countries, 2020-2023. Our World in Data, 2023.

By superimposing the phases of the pandemic (determined by the incidence of Covid-19) with the lethality curve (Fig 2), It was evident that the historical series of lethality were not stable and did not follow the historical series of cases. In Brazil, the highest lethality was recorded during phase 1 of the pandemic (8%), although the peak incidence of new cases of the disease occurred in phase 3 (1.2 million/week). In Russia, phase 2, which was one of the periods with the lowest number of cases, recorded the country’s second highest lethality during the pandemic (4.5%). In India, during phase 2, the highest peak in incidence that occurred between epidemiological weeks 09–19 of 2021 did not coincide with the peak in lethality, which occurred between weeks 43–50 of 2021. In China, the highest lethality (12%) occurred in phase 1. In contrast, phase 3 recorded the highest peak in incidence during the pandemic, with a lethality of around 2%. In South Africa, the highest incidence peak that occurred between epidemiological weeks 49 of 2021–02 of 2022 did not record the highest lethality, although it was high, reaching 7%. Furthermore, at the end of 2022, between epidemiological weeks 32–52, even with the steep drop in the incidence of Covid-19 in the country, the lethality reached around 4.5% (Table 1).

**Table 1:** Time trend parameters of historical fatality series in BRICS countries, 2020-2023.

Country		Phase	Period	APC	95% CI	P-value^a^
Brazil		1	EW 1|2020 to EW 12|2020	0.02	−0.03 to 0.07	0.767
			EW 12|2020 to EW 15|2020	79.29*	60.85 to 99.04	< 0.001
			EW 15|2020 to EW 20|2021	− 0.90	−6.47 to 9.25	0.482
			EW 20|2020 to EW 27|2020	− 12.53*	−21.71 to −9.15	0.020
			EW 27|2020 to EW 45|2020	− 1.15*	−2.16 to - 0.01	< 0.001
		2	EW 45|2020 to EW 49|2020	−8.84	−26.59 to 3.99	0.201
			EW 49|2020 to EW 14|2021	5.24	−2.15 to 18.52	0.051
			EW 14|2021 to EW 50|2021	−1.05*	− 1.88 to - 0.13	< 0.001
		3	EW 50|2021 to EW 03|2022	−47.85*	−54.84 to −41.93	< 0.001
			EW 03|2022 to EW 08|2022	38.05*	23.71 to 73.57	< 0.001
			EW 08|2022 to EW 21|2022	−2.96*	−6.10 to −0.25	0.002
		4	EW 21|2022 to EW 23|2022	−34.91*	−47.47 to −2.29	0.012
			EW 23|2022 to EW 33|2022	12.58*	8.45 to 42.18	0.004
			EW 33|2022 to EW 45|2022	1.95	−4.22 to 5.05	0.401
		5	EW 45|2022 to EW 18|2023	−1.30	−11.71 to 10.24	0.708
Russia		1	EW 1|2020 to EW 12|2020	1.92	−0.80 to 3.51	0.419
			EW 12|2020 to EW 14|2020	1983.04*	574.07 to 3457.61	< 0.001
			EW 14|2020 to EW 38|2020	3.51*	0.33 to 6.51	< 0.001
		2	EW 38|2020 to EW 41|2020	−9.79*	−15.03 to −6.48	< 0.001
			EW 41|2020 to EW 01|2021	2.26*	1.72 to 2.81	< 0.001
			EW 01|2021 to EW 10|2021	8.32*	7.35 to 9.70	< 0.001
			EW 10|2021 to EW 21|2021	0.15	−0.39 to 1.15	0.315
			EW 21|2021 to EW 23|2021	−7.71*	−11.13 to −2.30	0.029
		3	EW 23|2021 to EW 25|2021	−11.85*	−15.97 to −4.28	0.037
			EW 25|2021 to EW 36|2021	4.52*	3.71 to 5.79	0.006
			EW 36|2021 to EW 43|2021	−5.74*	−8.47 to −4.11	< 0.001
			EW 43|2021 to EW 02|2022	4.23*	3.39 to 5.25	< 0.001
		4	EW 02|2022 to EW 06|2022	−46.84*	−49.40 to −44.38	< 0.001
			EW 06|2022 to EW 11|2022	35.91*	30.68 to 44.92	< 0.001
			EW 11|2022 to EW 15|2022	13.02*	1.65 to 23.71	< 0.001
			EW 15|2022 to EW 26|2022	−1.22	−3.86 to 0.18	0.189
		5	EW 26|2022 to EW 33|2022	−29.49*	−32.64 to −27.02	< 0.001
			EW 33|2022 to EW 38|2022	1.05	−8.97 to 10.02	0.673
			EW 38|2022 to EW 43|2022	46.59*	35.58 to 58.97	< 0.001
			EW 43|2022 to EW 51|2022	−6.09*	−12.18 to −2.94	< 0.001
			EW 51|2022 to EW 01|2023	23.11*	0.87 to 35.78	0.036
		6	EW 01|2023 to EW 08|2023	−22.02*	−27.13 to −17.99	< 0.001
			EW 08|2023 to EW 18|2023	9.88*	6.60 to 14.14	< 0.001
India		1	EW 1|2020 to EW 12|2020	0.38	−0.16 to 0.90	0.334
			EW 12|2020 to EW 18|2020	8.38*	4.07 to 17.62	< 0.001
			EW 18|2020 to EW 22|2020	−10.69*	−19.75 to −2.68	0.003
			EW 22|2020 to EW 25|2020	14.65	−3.20 to 23.56	0.061
			EW 25|2020 to EW 31|2020	−14.57*	−24.17 to −2.88	0.018
			EW 31|2020 to EW 02|2021	−0.44	−1.08 to 1.38	0.674
			EW 02|2021 to EW 07|2021	−8.80*	−19.65 to −3.07	0.001
India	2		EW 07|2021 to EW 14|2021	−6.71	−24.47 to 2.28	0.156
			EW 14|2021 to EW 24|2021	21.60*	15.02 to 48.58	< 0.001
			EW 24|2021 to EW 36|2021	−10.13*	−17.84 to −6.31	< 0.001
			EW 36|2021 to EW 52|2021	12.72*	9.57 to 16.54	< 0.001
	3		EW 52|2021 to EW 02|2022	−76.16*	−87.25 to −19.89	0.069
			EW 02|2022 to EW 12|2022	45.71*	30.76 to 128.63	0.026
			EW 12|2022 to EW 22|2022	−20.11*	−32.01 to −9.54	< 0.001
	4		EW 22|2022 to EW 24|2022	−46.81	−63.49 to 6.55	0.264
			EW 24|2022 to EW 49|2022	9.22*	0.84 to 15.55	0.204
			EW 49|2022 to EW 18|2023	−7.61*	−10.84 to −4.63	< 0.001
China	1		EW 1|2020 to EW 22|2020	−42.75	−95.37 to 2.31	0.085
			EW 22|2020 to EW 29|2021	6.61	−29.54 to 234.32	0.056
			EW 29|2021 to EW 07|2022	−20.74*	−36.84 to −4.89	< 0.001
	2		EW 07|2022 to EW 14|2022	54.70*	44.46 to 66.00	< 0.001
			EW 14|2022 to EW 19|2022	−58.99*	−63.30 to −54.43	< 0.001
			EW 19|2022 to EW 25|2022	38.48*	25.72 to 60.28	< 0.001
			EW 25|2022 to EW 36|2022	−10.68*	−17.98 to −6.68	< 0.001
			EW 36|2022 to EW 49|2022	8.91*	5.33 to 14.15	< 0.001
	3		EW 49|2022 to EW 51|2022	−83.76*	−92.06 to −61.53	0.016
			EW 51|2022 to EW 02|2023	472.67*	210.39 to 777.56	< 0.001
			EW 02|2023 to EW 14|2023	−13.70*	−35.57 to −7.53	0.013
			EW 14|2023 to EW 18|2023	54.79*	5.21 to 231.96	0.016
South Africa	1		EW 1|2020 to EW 12|2020	2.89	−0.30 to 4.76	0.108
			EW 12|2020 to EW 15|2020	1571.31*	557.72 to 6524.51	< 0.001
			EW 15|2020 to EW 40|2020	2.89	−1.57 to 7.09	0.236
	2		EW 40|2020 to EW 44|2020	−42.24*	−67.58 to −25.02	< 0.001
			EW 44|2020 to EW 07|2021	17.14*	13.48 to 39.78	< 0.001
			EW 07|2021 to EW 13|2021	−6.51	−32.24 to 7.26	0.502
	3		EW 13|2021 to EW 25|2021	−10.81*	−15.19 to −7.42	< 0.001
			EW 25|2021 to EW 45|2021	10.69*	8.70 to 12.94	< 0.001
	4		EW 46|2021 to EW 49|2021	−70.01*	−78.49 to −58.91	< 0.001
			EW 49|2021 to EW 02|2022	88.73*	60.64 to 153.91	< 0.001
			EW 02|2022 to EW 07|2022	24.47	−21.69 to 45.37	0.403
			EW 07|2022 to EW 15|2022	−29.07*	−38.43 to −22.37	0.001
	5		EW 15|2022 to EW 18|2022	−32.49	−59.57 to 0.63	0.139
			EW 15|2022 to EW 26|2022	33.75*	22.25 to 94.35	0.023
			EW 15|2022 to EW 31|2022	−23.74*	−43.16 to −8.72	0.001
	6		EW 31|2022 to EW 49|2022	14.65	−36.12 to 108.76	0.246
			EW 31|2022 to EW 52|2022	−93.29	−97.60 to 155.55	0.437
			EW 31|2022 to EW 18|2023	−12.57	−36.98 to 156.21	0.481

**Legend:** EW – Epidemiological Week; APC – Annual Percentage Change; IC 95% CI – 95% confidence interval; ^a^ p-value estimated through ANOVA test. *Indicates that the Annual Percent Change (APC) is significantly different from zero at the alpha = 0,05 level. **Source:** Oue World in Data, 2023.

**Fig 3:**
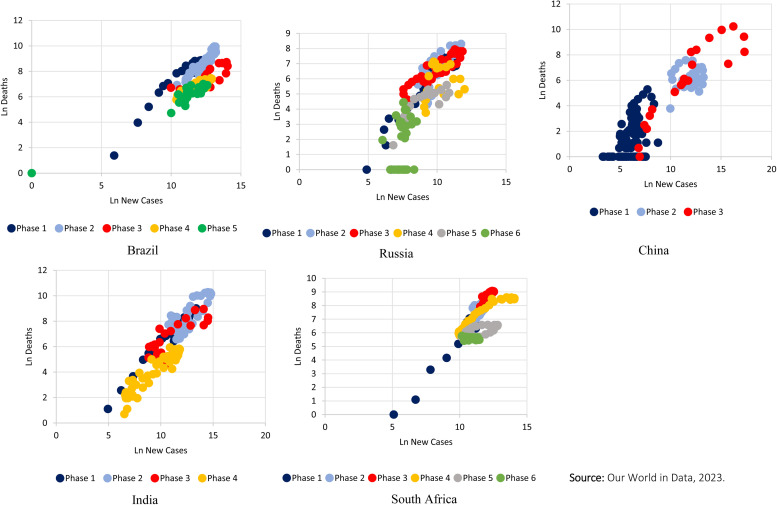
Correlation between new cases and new deaths from Covid-19 in BRICS countries (2020-2023). Our World in Data, 2023.

Over the 3 years of the pandemic, there have been important changes in relation to the emergence of new variants, the response capacity of health systems, the accumulation of knowledge about the disease and the ways to deal with it. Thus, a change was observed in the relationship between incidence and mortality from Covid-19 in the BRICS countries (Fig 3). In the case of Brazil, the strength of correlation between the two indices was very strong and presented a very discreet variation regardless of the phase (it varied between r = 0.9664; p-value < 0.001 and r = 0.8282; p-value < 0.001). In Russia, it is worth noting that during the last phase of the pandemic the correlation between incidence and mortality became negative (r = − 0.326; p value = 0.043), unlike what occurred in the previous phases. In India, the correlation gradually reduced during phases 1, 2 and 3, showing an increase in the last phase (r = 0.9337; p-value <0.001). In China, there was a sharp increase in correlation in the last phase of the pandemic (r = 0.9138; p-value < 0.001) and in South Africa, cyclical behavior was observed, with high correlation in odd-numbered phases and low correlation in even-numbered phases (Table 2).

**Table 2:** Correlation between indicators of incidence and mortality rates for Covid-19 (per 1 million inhabitants), BRICS countries, March/2020 to May/2023.

Countries	Brazil		Russia		India		China		South Africa	
Correlation Coeffcient	p-value^a^	Correlation Coeffcient	p-value^a^	Correlation Coeffcient	p-value^a^	Correlation Coeffcient	p-value^a^	Correlation Coeffcient	p-value^a^
Phase #1	0.9568	<0.001	0.9769	<0.001	0.9769	<0.001	0.4648	0.048	0.9372	<0.001
Phase #2	0.9306	<0.001	0.5509	0.034	0.7818	0.011	0.1938	0.613	0.7183	0.015
Phase #3	0.8282	<0.001	0.8447	0.002	0.8101	0.009	0.9138	<0.001	0.9191	<0.001
Phase #4	0.8938	<0.001	0.9564	<0.001	0.9337	<0.001	–		0.2533	0.583
Phase #5	0.9664	<0.001	0.6623	0.029	–		–		0.8120	0.008
Phase #6	–		− 0.3260	0.057	–		–		0.2313	0.602

**Legend:** ^a^ p-value estimated through Pearson’s correlation test.

## Discussion

When analyzing the pattern of lethality due to Covid-19 among the BRICS countries, it is observed that there were important differences related to conditioning factors in the context of each country. Brazil was the BRICS country to record the highest lethality from Covid-19 in the first phase of the pandemic, despite having a robust and well-structured health system, the Unified Health System (SUS). As possible conditioning factors, the absence of a mass testing policy for suspected cases, mild cases and contacts stands out, which would be fundamental in an active surveillance process to contain the transmission of Covid-19. For most of the period analyzed, the testing process prioritized symptomatic cases, first the most serious and then the moderate cases with typical symptoms, and remained concentrated in municipalities with greater service capacity. Added to this are the logistical difficulties in distributing tests and the political decision at different levels of government that also affected testing capacity [16].

In addition, the large inter-regional discrepancies in terms of health resources in Brazil stand out. According to data from February 2020 from the National Registry of Health Establishments (CNES), 90.4% of municipalities and 27.6% of the 450 health regions did not have resources to care for serious cases of Covid-19, such as ICU beds and respirators, especially in the North, Northeast and Central-West regions. This inequality resulted in long waiting lists for ICU admission and a high incidence of deaths due to lack of access, or late access to highly complex care [17]. Added to this context is the government’s denialist stance, which influenced the low levels of social distancing, impacting mortality from Covid-19, which certainly contributed to the increase in the lethality in the country [18].

The high lethality recorded by Russia during the early stages of the pandemic in a context of low numbers of registered cases can be attributed to the fact that at the beginning of the pandemic the country faced accusations of underreporting and low testing coverage. Since then, it has changed its strategy and adopted a mass testing policy, becoming one of the countries that has tested the most for Covid-19 in the world [19]. It is also worth noting that at the beginning of 2022, Russia experienced its most severe pandemic phase, associated with the spread of the BA.5 subvariant of Omicron, when it recorded the highest lethality during the pandemic. It is important to highlight that during this period the country had only around 50% of the population vaccinated. A study that analyzed mortality associated with the BA.5 subvariant in a comparative way, identified that the number of deaths in Russia was 3–5 times higher than in the United Kingdom, which in the same period had around 80% of its population vaccinated against Covid-19 [20,3].

In the case of India, during the first phase of the pandemic, the country had a low lethality of around 1%, possibly associated with low testing and underreporting of deaths. Regarding the first, by June 2022 the country had carried out about ¼ of the number of tests as Russia (609 and 2,034 tests per 1000 inhabitants respectively) [3]. Additionally, many Indian states have used rapid antigen detection tests, which are known to have a high percentage of false negatives. [21]. Regarding underreporting of deaths, although the Indian Council of Medical Research (ICMR) guidelines stipulated that confirmed or suspected Covid-19 deaths should be included in mortality data, there was no coordination from the Indian Ministry of Health and Family Welfare (MOHFW) regarding standardization of mortality registration across states. Furthermore, among the deaths recorded in the civil registration system, only 22% had a cause.

It is also worth noting that there was a delay in the lethality curve in India in relation to the incidence during phases 2 and 3 of the pandemic. This may be explained by the fact that the country faced difficulties in registering deaths in rural areas, where most of the population lives. In these areas, most deaths occurred outside of hospitals, leading to delays in registering deaths. [21].

China had the second highest lethality at the start of the pandemic compared to the BRICS, associated with the spread of the virus in the city of Wuhan and the probable underreporting of cases at the start of the health crisis. However, given the alarming situation in Wuhan, China implemented a package of timely strategies that included mass testing, the rapid establishment of a lockdown in both Wuhan and other cities in Hubei, as well as border controls on highways, train stations and airports in other regions of the country, and, subsequently, broad vaccination coverage. This response framework comprised the intersectoral and sectoral coordination instrument for the response to Covid-19 called the Joint Prevention and Control Mechanism, responsible for the country’s successful response in managing the health crisis [22,23].

It is worth noting, however, that despite the robust response to combat the pandemic during 2021, the country experienced a significant peak in lethality, possibly associated with the spread of the delta variant, particularly affecting regions such as Guangzhou, Shenzhen and Nanjing. This context was due both to the high transmissibility of the variant and the low vaccination coverage presented by the country at that time (around 40%) [24]. In this regard, other studies have also highlighted cases of post-vaccination SARS-CoV-2 infection associated with the delta variant [25,26].

It is important to highlight that, as vaccination progressed among countries, there was a downward trend in the number of hospitalizations, mortality and lethality due to Covid-19, even amid the circulation of variants of concern, as evidenced in several studies [27–29]. Paradoxically, South Africa recorded an increase in lethality at the end of 2022, associated with the low vaccination coverage of its population. A study that assessed the impacts of the Omicron variant on Covid-19 transmission, mortality and reinfection in South Africa and two other countries found that the unvaccinated population in these countries was, on average, three to four times more likely to die from Covid-19 complications, which was particularly alarming in the context of South Africa, which had a paltry vaccination coverage of 35% as of December 2022 [30,3].

The analysis of the pattern of lethality due to Covid-19 in the BRICS countries showed that there was no coordinated intra-BRICS response during the pandemic, but rather different approaches adopted according to the internal policy and capacity of each State, related to the production and acquisition of supplies such as tests and vaccines and flawed information systems. This reality contradicted what was agreed at the 12th, 13th and 14th annual Summits of the grouping, between 2020 and 2022, where the countries endorsed cooperation in the context of the Covid-19 pandemic, especially in relation to testing methods, therapeutics, research and production of vaccines, proposing, for example, the operationalization of the BRICS Vaccine R&D Center and the launch of the BRICS Vaccine Research and Development Center (CPDV/Brics) [31–33].

Among all the possibilities for intra-BRICS cooperation, the lack of vaccine diplomacy was the most emblematic example of the multilateral crisis in the bloc (which reflected a global crisis of multilateralism). Brazil has faced diplomatic difficulties with China after the Brazilian chief executive’s attacks on the country resulted in delays in the supply of Sinovac’s active pharmaceutical ingredient (API) to the Butantan Institute for the production of the vaccine. The India-China conflict over regional influence has prevented India from seeking help from China, although the latter has offered to share knowledge [34]. Furthermore, South Africa faced difficulties in acquiring vaccine doses, adding to the tragic scenario experienced by the African continent, in what was called by WHO Director General Tedros Adhanom as “Vaccine Apartheid” [35].

It is also worth noting that the quest to break the patent on vaccines, led by India and South Africa in October 2020, did not have the immediate endorsement of the other countries in the group. The remaining BRICS joined only six months later, offering their support in May and June 2021. Furthermore, even after the launch of the CPDV/BRICS in March 2022, there has been no concrete initiative for the development of a vaccine. [36]. In this regard, it is highlighted that the shared production of a single diplomatic vaccine in BRICS would represent a model of pandemic diplomacy and would provide faster and more equitable access to the vaccine for the Global South. However, the India-China conflict, the strained relationship between Brazil and China and distinct geopolitical and economic interests may have prevented the group from launching an immunizer with the BRICS “seal” [35].

It is worth noting that the lack of multilateral cooperation during the COVID-19 pandemic was also evident in other forums, such as the European Union, which experienced a lack of mutual support and solidarity among its Member States, delaying rapid action to deal with the pandemic crisis; the G20, which at its Global Health Summit held in 2021 did not establish ambitious commitments to address the impacts and lack of adequate responses to COVID-19 in developing and more vulnerable regions; and the G7, which did not discuss strategic issues that would have a major impact on overcoming the pandemic globally, such as a more equitable distribution of diagnostic tests, oxygen, hospital equipment and vaccines, at its summit held in June 2021 [37–39].

In this context, it is possible to affirm that the paradigm of global health security that determined the international response to the Covid-19 pandemic was contradictory to the central purpose of preparing for and responding to a global pandemic, since it requires multilateral action, and not autonomous national policies, guaranteeing the provision of global public goods that meet the needs of all people universally, as in the case of the vaccine. In this sense, the main lesson to be learned by the BRICS countries concerns diplomacy and global cooperation in health. For Petrone (2023), the potential and projection that BRICS has achieved in recent years make the group an alternative way for the reconstruction of multilateralism, potentially bringing greater democratization of decision-making processes to the world, promoting a plurality of visions and renewing Global Governance [40].

## Conclusions

The different patterns of lethality recorded by the BRICS countries throughout the pandemic, influenced by factors such as failures in testing capacity, in the death registration system and in access to vaccines, showed that the group did not achieve the expected performance at an extremely opportune moment to demonstrate to the world its differential as representatives of emerging powers, generating less impact, especially on the most vulnerable populations, present in all BRICS countries. One of the possible reasons for this scenario was the incipient articulation of BRICS as a multilateral forum, reflecting the weakening of global multilateralism. Thus, the need to strengthen multilateral cooperation to achieve more robust results in BRICS countries, such as improving vaccination rates, becomes evident. Furthermore, it is worth noting that the BRICS countries’ response scenario was also influenced by characteristics internal to each country, such as the structure of their health systems and political responses to mitigate the pandemic, such as social distancing and vaccination, requiring new studies on the reality of each case with a view to better understanding their response.

## Supporting information

S1 Data Study Database. (XLSX)

## References

[1] Gupta N, Singh B, Kaur J, Singh S, Chattu VK. Pandemia do covid-19 e reimaginação de multilateralismo através da diplomacia da saúde global. Sustentabilidade. 2021;13(20):11551. https://www.mdpi.com/2071-1050/13/20/11551

[2] Organização Pan-Americana da Saúde OPAS. Atualização epidemiológica: variantes de SARS-CoV-2 nas Américas. 2021. https://iris.paho.org/bitstream/handle/10665.2/53234/EpiUpdate26January2021_por.pdf?sequence=1&isAllowed=y

[3] Mathieu E, et al. Coronavírus Pandemia (COVID-19). https://ourworldindata.org/covid-vaccinations. 2023.

[4] Petrone F. Por que nós precisamos de um multilateralismo que funciona e o que é o papel de os BRICS: lições de o recente pandemia de Covid-19. Revista BRICS de Economia. 2023;4:35–51.

[5] Padula R, Fonseca F de CB da. BRICS: potencialidades de cooperação e papel na governança global de saúde no contexto da pandemia. Saúde debate. 2020;44(spe4):40–61. doi: 10.1590/0103-11042020e402

[6] Santos CA. Cooperação Sul-Sul, e o Multilateralismo Multinormativo na Criação do Centro de Pesquisa e Desenvolvimento de Vacinas do Brics. Revista Tempo do Mundo. 2023;31.

[7] Martins TC de F, Guimarães RM. Distanciamento social durante a pandemia da Covid-19 e a crise do Estado federativo: um ensaio do contexto brasileiro. Saúde debate. 2022;46(spe1):265–80. doi: 10.1590/0103-11042022e118

[8] Martins TC de F, Guimarães RM, Pereira AMM. BRICS in the management of the COVID-19 pandemic: a comparative study on social distancing and vaccination measures in the nations of the bloc. Cad Saude Publica. 2025;41(5):e00069024. doi: 10.1590/0102-311XPT069024 40498914 PMC12161504

[9] Frenk J, Godal T, Gómez-Dantés O, Store JG. A reinvigorated multilateralism in health: lessons and innovations from the COVID-19 pandemic. Lancet. 2022;400(10363):1565–8. doi: 10.1016/S0140-6736(22)01943-2 36216020 PMC9544940

[10] Preston S, Heuveline P, Guillot M. Demography: measuring and modeling population processes. Malden, Massachussets: Blackwell Publishers; 2001.

[11] Kim H, Fay MP, Feuer EJ, Midthune DN. Permutação testes para joinpoint regressão com formulários para taxas de câncer. Estatisticas em Medicina. 2000;19:335–35. https://pubmed.ncbi.nlm.nih.gov/10649300/

[12] Vieira S. Introdução à bioestatística. 4 ed. Rio de Janeiro: Elsevier. 2008.

[13] Martins-Filho P, et al. Socio-econômico desigualdades e incidência de COVID-19 e mortalidade no Brasil crianças: um cenário nacional baseado em registro estudar. Saúde Pública. 2021.

[14] Kim H, Fay MP, Feuer EJ, Midthune DN. Vinte anos desde Joinpoint 1.0: duas melhorias principais, suas justificativa, e impacto. Stat Med. 2022;41:3102–30.35522060

[15] Conselho Nacional de Saúde Brasil. Resolução nº 466, de 12 de dezembro de 2012. Aprova diretrizes e normas reguladoras de pesquisas envolvendo seres humanos. 2012. https://bvsms.saude.gov.br/bvs/saudelegis/cns/2013/res0466_12_12_2012.html

[16] Machado CV, Pereira AMM, Freitas AMM, editors. Políticas e sistemas de saúde em tempos de pandemia: nove países, muitas lições. Série Informação para ação na Covid-19 | Fiocruz. 2022. doi: 10.7476/9786557081594

[17] Fiocruz. Boletim Observatório Covid-19. Balanço de dois anos da pandemia Covid-19. Janeiro de 2020 a janeiro de 2022. Observatório Covid-19/ Fiocruz. 2022. https://portal.fiocruz.br/sites/portal.fiocruz.br/files/documentos_2/boletim_covid_2022balanco_2_anos_pandemia-redb.pdf

[18] Siqueira CADS, de Freitas YNL, Cancela M de C, Carvalho M, da Silva LP, Dantas NCD, et al. COVID-19 in Brazil: trends, challenges, and perspectives after 18 months of the pandemicCOVID-19 en Brasil: tendencias, desafíos y perspectivas después de 18 meses de pandemia. Rev Panam Salud Publica. 2022;46:e74. doi: 10.26633/RPSP.2022.74 35875320 PMC9299398

[19] Garcia AS, Curty R, Aguiar AC, Rezende L, Dantas MV. Os BRICS frente à pandemia da COVID-19: uma análise preliminar sobre políticas comparadas. CI. 2021;17(3):33–46. doi: 10.5752/p.1809-6182.2020v17n3p33-46

[20] Loseva P. Preocupado com o guerra, Rússia ignorado um aceno de cobiça e ignorado outro. BMJ. 2022;379. https://www.bmj.com/content/bmj/379/bmj.o2825.full.pdf

[21] Chatterjee P. É Índia faltando mortes de COVID-19?. Lancet. 2020. https://www.ncbi.nlm.nih.gov/pmc/articles/PMC7470692/

[22] Pereira A. A resposta à Covid-19 na China: planejamento central e governança nacional da vigilância e atenção à saúde. In: Machado CV, Pereira AM, Freitas CM, editors. Políticas e sistemas de saúde em tempos de pandemia: nove países, muitas lições. Rio de Janeiro, RJ: Observatório Covid-19 Fiocruz. 2022.

[23] Zhou Y, et al. Uso de contato rastreamento, isolamento e massa testando para ao controle transmissão da covid-19 na China. BMJ. 2021;375. https://www-bmj-com.ez68.periodicos.capes.gov.br/content/375/bmj.n2330

[24] Xiang B, et al. Características e manejo do SARS-CoV-2 delta infecções por COVID-19 induzidas por variantes de maio a Outubro de 2021 na China: pós-vacinação casos de infecção. Am J Transl Res. 2022;14(6):3603–9. https://www.ncbi.nlm.nih.gov/pmc/articles/PMC9274556/35836857 PMC9274556

[25] Graham MS, Sudre CH, May A, Antonelli M, Murray B, Varsavsky T, et al. Changes in symptomatology, reinfection, and transmissibility associated with the SARS-CoV-2 variant B.1.1.7: an ecological study. Lancet Public Health. 2021;6(5):e335–45. doi: 10.1016/S2468-2667(21)00055-4 33857453 PMC8041365

[26] Antonelli M, Penfold RS, Merino J, Sudre CH, Molteni E, Berry S, et al. Risk factors and disease profile of post-vaccination SARS-CoV-2 infection in UK users of the COVID Symptom Study app: a prospective, community-based, nested, case-control study. Lancet Infect Dis. 2022;22(1):43–55. doi: 10.1016/S1473-3099(21)00460-6 34480857 PMC8409907

[27] Chemaitelly H, et al. Vacina mRNA-1273 COVID-19 eficácia contra as variantes B.1.1.7 e B.1.351 e doença grave de COVID-19 no Catar. Medicina Natural. 2021;27:1614–21. https://pubmed.ncbi.nlm.nih.gov/34244681/10.1038/s41591-021-01446-y 34244681

[28] Haas E, et al. Impacto e eficácia da vacina mRNA BNT162b2 contra infecções por SARS-CoV-2 e casos, hospitalizações e mortes de COVID-19 após um vacinação campanha em Israel: uma observacional estudar usando nacional dados de vigilância. The Lancet. 2021;397:1819–29. https://www.thelancet.com/article/S0140-6736(21)00947-8/fulltext

[29] Nasreen S, et al. Eficácia das vacinas COVID-19 contra a infecção sintomática por SARS-CoV-2 e forte resultados com variantes de preocupação em Ontário. Natureza Microbiologia. 2022;7:379–85. https://pubmed.ncbi.nlm.nih.gov/35132198/

[30] Xavier C, et al. Caracterização de variante Omicron durante a pandemia de COVID-19 e o impacto de vacinação, taxa de transmissão, mortalidade e reinfecção na África do Sul, Alemanha e Brasil. Biotecnologia. 2022;11. https://www.mdpi.com/2673-6284/11/2/12

[31] Brasil M das RE. Declaração de Moscou da XII Cúpula do BRICS. https://www.gov.br/mre/ptbr/canais_atendimento/imprensa/notas-a-imprensa/2020/declaracao-de-moscou-da-xii-cupulado-brics. 2020. Accessed 2023 September 2.

[32] Brasil, Ministério das Relações Exteriores. XIII Cúpula do BRICS – Declaração de Nova Delhi. https://www.gov.br/mre/ptbr/canais_atendimento/imprensa/notas-a-imprensa/xiii-cupula-brics-declaracao-de-novadelhi. 2021. Accessed 2023 September 2.

[33] Brasil. Ministério das Relações Exteriores. Declaração de Pequim da XIV Cúpula do BRICS. https://www.gov.br/mre/ptbr/canais_atendimento/imprensa/notas-a-imprensa/declaracao-de-pequim-da-xiv-cupuladobrics#:~:text=Conclamamos%20a%20comunidade%20internacional%20a,%2C%20sustent%C3%A1vel%2C%20equilibrada%20e%20inclusiva. 2022. Accessed 2023 September 2.

[34] Hoirisch C. Quo vadis, Brics? Colaboração biofarmacêutica, diplomacia vacinal dos BRICs e (des)motivações para o cumprimento dos compromissos acordados sobre vacinas Covid-19. In: Buss P, Burger P, editors. Diplomacia da saúde: respostas globais à pandemia. Fiocruz. 2021. p. 317–28. https://www.arca.fiocruz.br/handle/icict/50463

[35] Mundo vive apartheid de vacinas contra Covid-19, diz diretor da OMS. G1. 2021. https://g1.globo.com/bemestar/vacina/noticia/2021/05/17/mundo-viveevacinascontracov

[36] Moore C. Os BRICS e a diplomacia da saúde global na pandemia de Covid-19: situando a diplomacia dos BRICS dentro de o saúde global prevalecente governança contexto. Revista Brasileira de Política Internacional. 2022;65.

[37] Akon MdS, Rahman M. Reshaping the Global Order in the Post COVID-19 Era: A Critical Analysis. Chin J Int Rev. 2020;02(01):2050006. doi: 10.1142/s2630531320500067

[38] Alcázar S, Buss PM. Sobre G-7 e avalanches pandêmicas. In: Buss P, Burger P, editors. Diplomacia da saúde: respostas globais à pandemia. Rio de Janeiro: Fiocruz. 2021.

[39] C20 First Reaction to the Rome Declaration of Principles Released by the Global Health Summit. https://civil-20.org/c20-first-reaction-to-therome-declaration-of-principles-released-by-the-global-health-summit/. 2021. Accessed 2021 September 30.

[40] Fukuda-Parr S, Buss P, Ely Yamin A. Pandemic treaty needs to start with rethinking the paradigm of global health security. BMJ Glob Health. 2021;6(6):e006392. doi: 10.1136/bmjgh-2021-006392 34083245 PMC8182745

