# Correction: Comparative analysis of the lethality pattern of Covid-19 and the response of BRICS countries to the pandemic in the context of multilateralism: An ecological study

**DOI:** 10.1371/journal.pone.0340478

**Published:** 2026-01-05

**Authors:** Thalyta Cássia de Freitas Martins, Adelyne Maria Mendes Pereira, Cristiani Vieira Machado, Carlos Machado de Freitas, Raphael Mendonça Guimarães

The following information is missing from the Funding statement: This activity was promoted by the Postgraduate Program in Public Health of the National School of Public Health (ENSP/Fiocruz), with funding from the Academic Excellence Program of the Coordination for the Improvement of Higher Education Personnel (CAPES PROEX) (no. 3657/2025/88881.192090/2025-01). Funding was granted to the author Guimarães, RM.
